# Endothelial cells promote metastasis of prostate cancer by enhancing autophagy

**DOI:** 10.1186/s13046-018-0884-2

**Published:** 2018-09-10

**Authors:** Ruizhe Zhao, Xiaoyu Bei, Boyu Yang, Xiaohai Wang, Chenyi Jiang, Fei Shi, Xingjie Wang, Yiping Zhu, Yifeng Jing, Bangmin Han, Shujie Xia, Qi Jiang

**Affiliations:** Department of Urology, Shanghai General Hospital, Shanghai Jiao Tong University School of Medicine, 85 Wujin Road, Shanghai, 200080 NO China

**Keywords:** Endothelial cells, Autophagy, Androgen receptor, Metastasis

## Abstract

**Background:**

Prostate cancer is one of the most common malignancies. Increasing evidence suggested that endothelial cells may contribute to prostate cancer progression and metastasis. Most recently, autophagy has been proposed to plays a significant role in tumorigenesis and metastasis. Also, it is reported that downregulation of androgen receptor (AR) induces autophagy in prostate cancer cells. However, the underlying mechanisms remain unclear. Here, we aim to explore the role and mechanisms of endothelial cell in prostate cancer progression.

**Methods:**

The coculture system was established to test the effect of endothelial cells on prostate cancer cells. We performed antibody array and ELISA were used to profile the cytokine expression pattern of endothelial cells in supernatant. Western blot and RT-PCR were used to determine the mechanism by endothelial cells to promote invasion ability of prostate cancer cells. Maraviroc and chloroquine were used to block the CCL5/CCR5 and autophagy pathway respectively. Orthotopic xenograft mouse models and drug treatment study were conducted to determine the role of endothelial cells in promoting metastatic potential in vivo.

**Results:**

We use CPRC prostate cancer model and demonstrate that endothelial cells secrete large amount of CCL5 and induces autophagy by suppressing AR expression in prostate cancer cell lines. Consequently, elevated autophagy accelerates focal adhesions proteins disassembly and promoted prostate cancer invasion. Inhibition of both CCL5/CCR5 signaling and autophagy significantly reduces metastasis in vivo.

**Conclusions:**

Together, our data establish the function for endothelial cells in tumor metastasis and propose new drug target for mCRPC.

**Electronic supplementary material:**

The online version of this article (10.1186/s13046-018-0884-2) contains supplementary material, which is available to authorized users.

## Background

Prostate cancer is one of the most common malignancies and causes the second leading cancer related death in males worldwide [1]. Most prostate cancer cases are initially localized and grow slowly. Usually it takes years to develop into advanced disease. These patients are hormone-sensitive and are treated with hormone therapy, also called androgen-deprivation therapy (ADT) or androgen suppression therapy, which is the first line treatment for prostate cancer [2]. Despite early success in suppressing prostate tumor growth, most tumors will eventually develop resistant to hormone therapy, leading to tumor recurrence and the disease becomes castration resistant prostate cancer (CRPC). CRPC tumors expand outside the prostate into adjacent areas or by moving to distant organs through the blood flow, eventually entering the lethal stage called metastatic castration resistant prostate cancer (mCRPC). Notably, only about 27% of mCRPC patients survive in 5 years [1].

Cancer metastasis is a multi-step process of complex, interrelated events including detachment, migration, invasion and adhesion [3]. Tumor microenvironment (TME) composed of parenchyma, nonmalignant cells (inflammatory cells, cancer-associated fibroblasts, angiogenic vascular cells, and sometimes adipocytes) and extracellular matrix constitute the stromal [4], have been reported implicated in prostate cancer metastasis. Increasing evidence suggested that endothelial cells may contribute to prostate cancer progression and metastasis. In response to ADT, the prostatic microvascules will go through apoptosis but regenerated rapidly in CRPC [5]. And increased infiltration of microvascules in tumor promotes distal metastasis of CRPC, partly through AR signaling [6, 7]. These results emphasize the importance of endothelial cells in prostate cancer metastasis.

Autophagy is a genetically programmed, evolutionarily conserved process plays a homeostatic role in normal cells. It is primarily regulated in a post-translational manner to permit a rapid response to nutrient stress cross all eukaryotic cells [8]. The autophagic flux is defined as formation and maturation of the autophagosomes and its fusion with the lysosomes, degradation of cargo and release of macromolecules into the cytosol [9]. The role of autophagy in prostate cancer is still controversial. The biopolar effect of autophagy may vary according to the stage of disease. In early stages, the induction of autophagy may increase the cell death [10] but the late stage of prostate cancer may take advantage of autophagy to reduce the damage of chemotherapy drugs or meet the requirements necessary for tumor survival and rapid proliferation [11, 12]. The role of autophagy in promoting cancer metastasis has been revealed in the recent studies.

Till now, few studies were focused on the relationship of endothelial cells, autophagy and cancer metastasis. In this study, by multiple in vitro and in vivo strategies, we tried to build a bridge of endothelial cells induced autophagy and metastasis in CRPC, and to identify regulators of metastasis for new therapeutic targets and agents to benefit the treatment of CRPC.

## Methods

### Cell lines and treatment

Human umbilical vein endothelial cells (HUVEC) was obtained from the American Type Culture Collection (ATCC, VA, USA) and maintained in Dulbecco’s Modified Eagle Medium supplemented with 10% growth factors (ATCC). CWR22Rv-1 was obtained from the Chinese Academy of Sciences Committee on Type Culture Collection Cell Bank (Shanghai, China). C4–2 was obtained from the American Type Culture Collection. Both 22RV-1 and C4–2 were cultured in RPMI 1640 medium with 10% fetal bovine serum and 1% penicillin-streptomycin. Cells were cultured in 37 °C and 5% of CO2 in humidified air. Sixwell (3 mm) transwell plates (Corning, NY, USA) were used for coculture. Chloroquine (Selleckchem, TX, USA), rapamycin(Sigma-Aldrich, NY, USA) and CCL5 (Peprotech, NJ, USA) were used for autophagy regulation.

### Cytokine profile with human cytokine microarray and ELISA assay

A commercial quantitative microarray (Human Inflammation Antibody Array G-Series 3, RayBiotech, GA, USA) was used to profile the cytokine expression pattern in the cell supernatant. Each experiment was carried out in accordance with the manufacturer’s instructions. The glass chips were first incubated with blocking buffer at room temperature for 30 min. Then blocking buffer was carefully removed and the chips were overlaid with 100 mL of diluted sample. After 2 h incubation at room temperature, gently washed the chips with wash buffers. About 70 μl of 1X biotin-conjugated anti-cytokines were added to each subarray and then washed away, followed by incubation with Streptavidin - HiLyte Plus™ Fluor 555. The signals (532 nm excitation) were scanned and extracted using InnoScan 300 Microarray Scanner (Innopsys, Inc. France). The results were analyzed using the RayBiotech Q Analyzer program.

Cell culture media was collected 24 h after cocultured with or without HUVEC. C-C motif chemokine ligand 5 (CCL5) level was determined using a commercial human CCL5 ELISA kit (RayBiotech, GA, USA) according to the manufacturer’s instructions.

### RNA extraction and quantitative real-time PCR (qRT-PCR)

Total RNA was extracted using Trizol (Invitrogen) according to the manufacturer’s instruction. RNA integrity was evaluated with electrophoresis using an agarose gel (1%) stained by ethidium bromide (Sigma). cDNAs were synthesized with the Prime-Script RT reagent kit (Takara, Dalian, China). To determine the gene expression levels, the RT-QPCR reaction was prepared using quantitative polymerase chain reaction (qPCR) was performed using SYBR® Premix Ex Taq™ II PCR kit (Takara, Dalian, China). The primers used for reverse transcription and qPCR are summarized in Additional file 1: Table S1. GAPDH was used as an internal control. The relative mRNA levels of the target genes were normalized to GAPDH by using the 2-ΔΔCq method.

### In vitro cell invasion assays

A total of 2.5 × 10^4^ cells suspended in 200 μL of serum-free medium were seeded in the upper Transwell chamber BioCoat™ Matrigel Invasion Chamber (Corning LifeSciences, NY, USA) plated into 24-well plates. Medium with 20% FBS was added into each lower chamber. After 24 h incubation, the membranes were fixed in 4% paraformaldehyde and stained with 0.1% crystal violet (Yeasen, Shanghai, China). The invaded cells were counted in five randomly selected fields under microscopy, and the average value was calculated. Each experiment was conducted in triplicate.

### Western blot and antibodies

Cells were lysed in a RIPA lysis buffer with protease inhibitor cocktail. A total of 20 μg of protein was separated by 10–15% gradient SDS-polyacrylamide gel electrophoresis (Beyotime, Shanghai, China) and transferred to polyvinylidene fluoride membranes (Immobilon-P; Millipore, Darmstadt, Germany). After blocking with 10% milk for 1 h, the blocked membranes were incubated with primary antibodies at 4 °C overnight. Appropriate secondary antibodies conjugated with horseradish peroxidase and Pierce ECL Western Blotting Substrate (Thermo Fisher Scientific, NY, USA) were used to detected target proteins by ChemiDoc™ XRS+ System (Bio-rad, CA, USA).

Western blot was carried out using the following antibodies: anti-AR (Cell Signaling Technology #3202, rabbit monoclonal, 1:1000 dilution), anti-GAPDH (Sangon #D110016, rabbit polyclonal, 1:4000 dilution), anti-Beclin-1 (Cell Signaling Technology #3495, rabbit monoclonal, 1:1000 dilution), anti-Atg5 (Cell Signaling Technology # 12994, rabbit monoclonal, 1:1000 dilution), anti-LC3A/B (Cell Signaling Technology #12741, rabbit monoclonal, 1:1000 dilution), anti- anti-SQSTM1/p62 (Abcam #ab91526, rabbit polyclonal, 1:1000 dilution), anti-Paxillin (Abcam # ab32084, rabbit monoclonal, 1:1000 dilution), anti-Zyxin (Abcam # ab50391, monoclonal, 1:1000 dilution). All images are representative of a minimum of three independent experiments.

### siRNA transfection

Transfection was achieved using Lipofectamine 2000 Transfection Reagent (Thermo Fisher Scientific, NY, USA) according to the manufacturer’s protocol. Briefly, cells were seeded at a concentration of 1 × 10^5^ cells/well in 6-well culture plates. After plating for 24 h, the transfection was performed with specific siRNA or non-targeting siRNA for 6 h in OptiMEM media. After transfection, cells were washed twice with PBS and cultured in regular condition and used for experiments at 24 h. Sequences of siRNA are summarized in Additional file 1: Table S1.

### GFP-LC3 puncta assay

Autophagy was examined by analyzing the formation of fluorescent puncta of autophagosomes in cells transfected with GFP-LC3. Cells were transfected with 2 μg/ml GFP-LC3 plasmid in six-well plates according to the manufacturer’s protocol. After transfection, the cells were treated with different conditions. Image acquisition was performed using a fluorescence microscope.

### Immunofluorescence assays

Cells were grown on sterile slide in 24-cm cell culture plates and allowed to attach by overnight incubation, then washed with PBS, followed by fixation with 4% paraformaldehyde and permeabilization with 0.1% Triton X-100. After incubated with blocking solution and then treated with primary antibodies, the cells were incubated with fluorescein-labeled secondary antibodies. The stained slides were sealed with anti-fade mounting medium and visualized with fluorescence microscopy.

### Luciferase reporter assay

A total of 10,000 prostate cancer cells were seeded in a 24-well plate and Lipofectamine 2000 (Invitrogen) was used according to the manufacturer’s protocol. Hundred nanograms of mouse mammary tumor virus (MMTV)-luc containing androgen response element (ARE) sequence were transfected 24 h before assessment of luciferase. Firefly and Renilla luciferase were measured with Dual Luciferase Assay (Promega). Data are shown as relative light units and representative of at least two independent experiments. Firefly luciferase is normalized for Renilla luciferase.

### In vivo animal studies and in vivo bioluminescence image

All animal studies were carried out in compliance with guidelines of the Chinese Council on Animal Care. Protocols were approved by the Medical Science Ethics Committee of Shanghai General Hospital. High metastatic C4–2 prostate cancer cell lines were transfected with CMV-RFPT2A-Luciferase Lentivirus (Genomeditech, Shanghai, China). Orthotopic tumors were induced by cell injection within the prostate on anesthetized male BALB/c-nude mice (6–8 weeks). Cells (5 × 10^5^/10 μL per lobe) suspended in 20 μL 50% matrigel were injected in the two dorsal prostate lobes. Prostate tumors were monitored by IVIS Imaging System (Xenogen Technology, AZ, USA) every week. All fluorescence images were acquired with a 25 s exposure. Images and measurements of bioluminescent signals were acquired and analyzed using Living Image software (Xenogen Technology). The end-point of the experiment was day 95, and remaining mice still alive were euthanized by cervical dislocation.

### Hematoxylin and eosin staining and Immunohistochemical analysis

Tumors were resected in 2-μm thickness and fixed in 4% paraformaldehyde, embedded with paraffin. The cross-sectioned tissues were stained with H&E to observe histology. For immunohistological analysis, paraffin sections were dewaxed in xylene and rehydrated in graded ethanol, followed by incubation with non-specific protein blocking solution 1% bovine serum albumin (Thermo Fisher Scientific, # 11021037;, Waltham, USA) in PBS for 45 min at room temperature, and incubated with primary antibodies against AR (1:300, Abcam, ab133273, Cambridge, UK) or Paxillin (1:400, Abcam, ab32084) overnight at 4 °C. For negative controls, blocking solution was added instead of the primary antibody. Then the slides were incubated with EnVision-HRP secondary antibody for 1 h. a. The slides were developed with diaminobenzidine detection kit (Dako cytomation, Denmark). After being counterstained with haematoxylin, the samples were visualized under a light microscope (Olympus, Tokyo, Japan).

### Statistical analysis

All data were expressed as mean ± standard derivation (S.D.) of three independent experiments, as indicated. Statistical analysis was performed using SPSS software package (version16.0, SPSS Inc). For parametric analyses, 2-tailed Student’s t test or one-way ANOVA was used. For nonparametric analyses, Mann-Whitney U test was used. *P* < 0.05 was considered statistically significant.

## Results

### Endothelial cells enhances invasion of prostate cancer cells through downregulation of androgen receptor

**Fig. 1:**
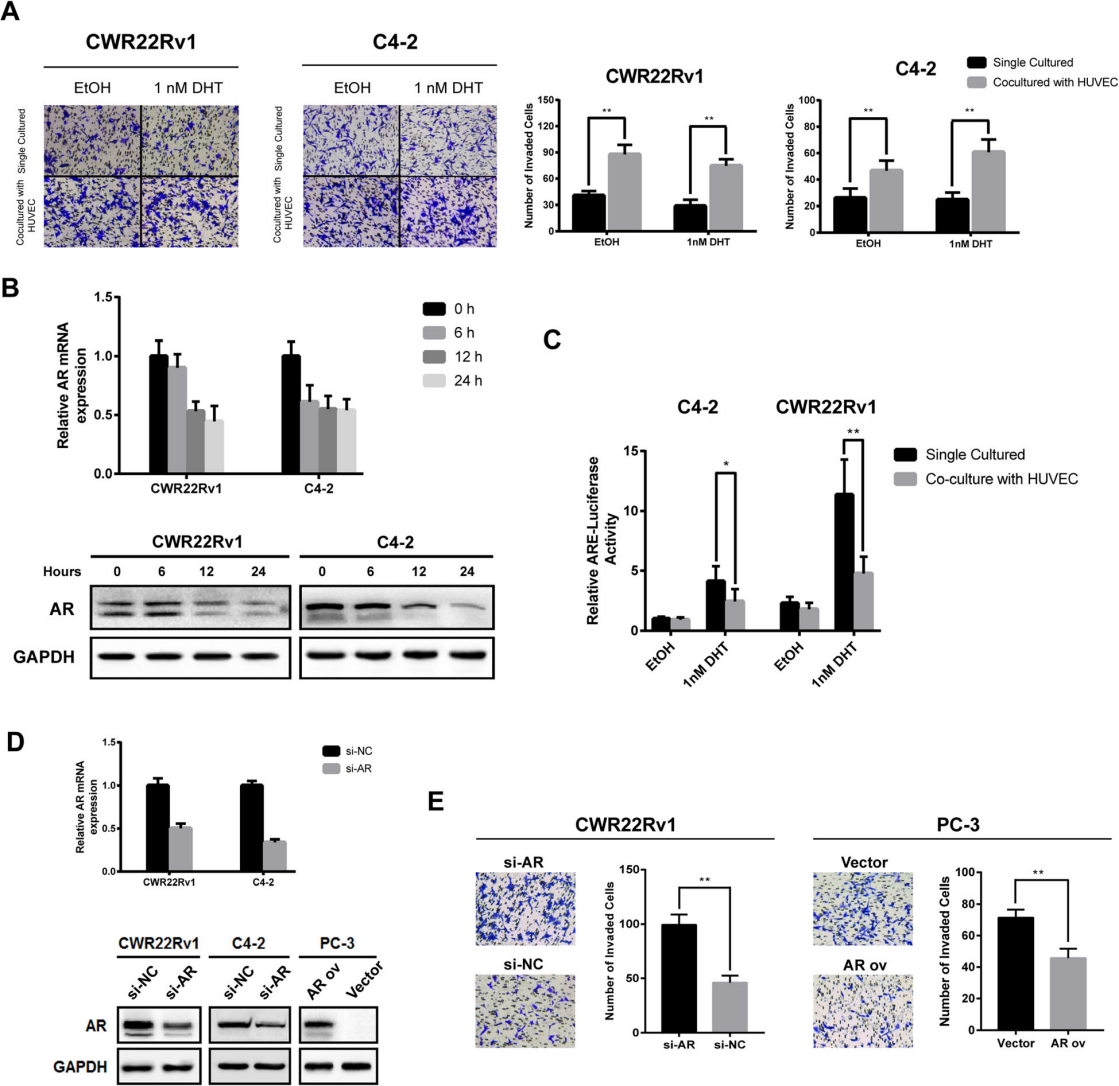
Endothelial cells promote invasion of prostate cancer cell by suppressing AR. **a** Transwell invasion assays of CWR22Rv1 and C4–2 with or without HUVEC. **b** C4–2 and CWR22Rv1 cells were cocultured with HUVEC for 6, 12, 24 h. Quantitative PCR and western blot analysis were performed to assess AR expression. **c** C4–2 and CWR22Rv1 cells were transfected with MMTV-luc containing ARE and cocultured with HUVECs (medium for control) in the presence of 1 nM DHT as indicated. After 24 h, luciferase activity was measured. **d** C4–2 and CWR22Rv1 cells were transfected with siRNA targeting AR and PC-3 cell was overexpressed with AR, western blot was conducted to assess AR expression. **e** Transwell invasion assay of CWR22Rv1 and PC-3 cells with or without AR manipulation

The coculture system was established to test the effect of endothelial cells on prostate cancer cells. CWR22Rv1 and C4–2 were cocultured with endothelial cell HUVEC separately. As is Fig. 1a indicated, the invasion ability of both CWR22Rv1 and C4–2 were enhanced with the existence of HUVEC, through cell viability was not affected (Additional file 2: Figure S1). Previous study has revealed that endothelial cells promoted invasion of prostate cancer cells by suppressing AR signaling. We examined the AR expression in the coculture system. Time course realtime qPCR and western blot both confirmed that AR was downregulated as early as 6 h, indicating AR alteration is prior to invasion increasing (Fig. 1b). ARE-driven luciferase assay was also performed and showed that AR transcription activity decreased after coculturing (Fig. 1c). We further added 1 nM dihydrotestosterone (DHT), which is potent agonist of AR, into the coculture system. As expected, ARE luciferase activity of both C4–2 and CWR22Rv1 cells was significantly elevated instantly after DHT treatment (Fig. 1c), whereas endothelial cells partially blocked the effect of DHT and enhanced invasion ability of cells. These results suggested endothelial cells can affect both expression and activity of AR. To further explore the role of AR in CRPC cells, we knocked down the expression of AR by transfecting siRNA targeting AR in C4–2 and CWR22Rv1 or overexpressing AR in AR negative PC-3 cells (Fig. 1d). AR knockdown significantly increased CWR22Rv1 cell invasion, and it is also true for C4–2, as PC-3-AR cells loses invasion ability compared with parental PC-3 cells (Fig. 1e). Together, results from Fig. 1 suggested that endothelial cells enhances invasion of prostate cancer cells through suppressing AR.

### CCL5 released by endothelial cells promotes invasion of prostate cancer cells by suppressing AR expression

**Fig. 2:**
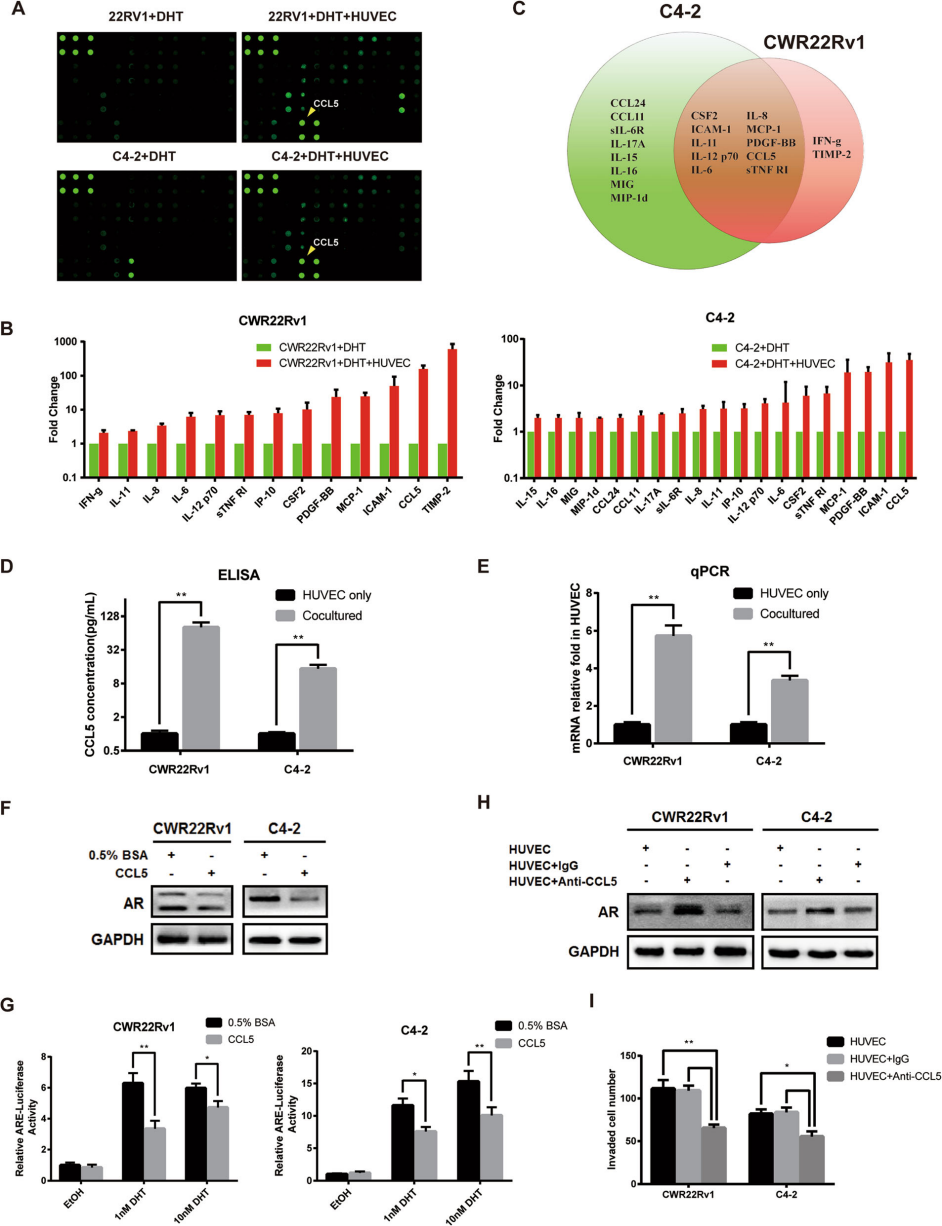
CCL5 secreted by endothelial cells mediates AR decrease in CWR22Rv1 and C4–2 cells and enhances cell invasion. **a** Cytokine array analysis. CWR22Rv1 or C4–2 cells were cocultured with or without HUVEC cells for 2 days and the conditioned media collected for cytokine array analysis. **b** & **c** Cytokines that differentially secreted in conditioned media between two conditions were analyzed. **d** CCL5 ELISA assay for CCL5 level in conditioned media **e**. Quantitative PCR analysis of CCL5 in HUVEC. **f** CWR22Rv1 or C4–2 cells (1 × 10^5^/well) were treated with 20 ng/mL of human recombinant CCL5 (Peprotech) for 6 h, then western blot analysis was performed for AR expression. **g** CWR22Rv1 or C4–2 cells were transfected with MMTV-luc containing ARE. After transfection, cells were treated with 20 ng/mL of CCL5 for 24 h in the presence of DHT as indicated, and luciferase activity was measured. **h** & **i** C4–2 and CWR22Rv1 cells were cocultured with HUVECs in the presence of anti-CCL5–neutralizing antibody (R&D Systems) for 36 h, invasion assay or western blot analysis was conducted

Endothelial cells were reported to produce tumor-promoting factors that stimulate progression of prostate cancer. We seek to find out the critical factor that may promote prostate cancer metastasis. According to manufacturer’s instructions, we conducted cytokine array to profile the cytokine expression pattern in presence or absence of HUVEC in the coculture system (Fig. 2a). Compared with C4–2 or CWR22Rv1 single culturing, several chemokines/cytokines were significantly increased when cocultured with HUVEC, among which CSF2, ICAM-1, IL-11, IL-6, IL12 p70, IL-8, MCP-1, PDGF-BB, CCL5 and sTNF R1 were elevated in both cocultrue systems (Fig. 2b, c). We noticed that CCL5 was most secreteed factor by endothelial cells. Extensive studies on CCL5 and its receptor C-C motif chemokine receptor 5 (CCR5) indicated that CCL5 may play an important role in tumor progression in hematological malignancies, lymphomas, and a great number of solid tumors [13]. ELISA analysis of culture media confirmed that CCL5 was increased (Fig. 2d). Also, analysis of CCL5 expression in HUVEC showed that CCL5 mRNA increased significantly (Fig. 2e). And knocking down CCL5 by small interfering RNA in HUVEC reduced CCL5 secretion in culture media (Additional file 3: Figure S2A &B), indicating endothelial cells are main source of the secretory CCL5 and potential cell to cell interaction between prostate cancer cells and endothelial cells. To further study the effect of CCL5 on prostate cancer cells and AR, we treated C4–2 or CWR22Rv1 with CCL5 respectively. Western blot and luciferase activity analyses both showed that CCL5 treatment could reduce AR expression (Fig. 2f) and AR transactivation, even in presence of DHT (Fig. 2g). Reducing the concentration of active CCL5 in media by using CCL5 neutralizing antibody reversed effects of HUVEC on AR downregulation and invasion enhancement. Together, these results indicate that CCL5 released by endothelial cells may be the essential factor to induce AR downregulation and consequently increase invasion ability of prostate cancer.

### Endothelial cells induce autophagy that results in enhanced prostate cancer cell invasion by suppressing AR

**Fig. 3:**
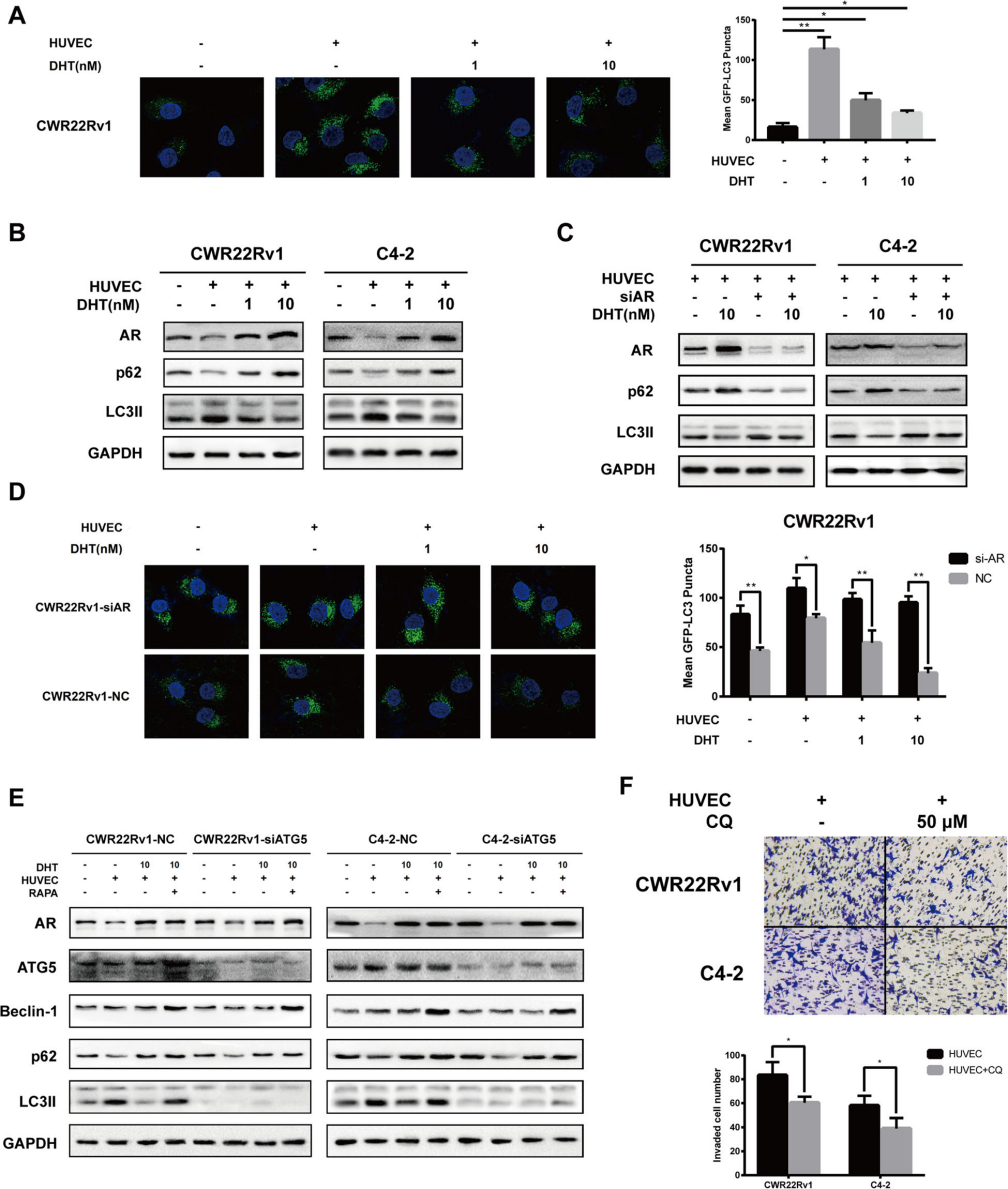
Endothelial cells induce autophagy by repressing AR. **a** Autophagosomes were detected by immunofluorescence for LC3-GFP in CWR22Rv1 cell in presence of HUVEC and/or DHT. **b** Western blot analysis of AR, p62, LC3II in presence of HUVEC and/or DHT. **c** Western blot analysis of AR, p62, LC3II in AR knockdown or parental cells in presence of HUVEC and/or DHT. **d** Autophagosomes were detected by immunofluorescence for LC3-GFP in CWR22Rv1 or AR knockdown CWR22Rv1 in presence of HUVEC and/or DHT. **e** Western blot analysis of AR, Atg5, Beclin-1, p62, LC3II. **f** C4–2 and CWR22Rv1 cells were cocultured with HUVECs in the presence or absence of CQ (Sigma) for 36 h, invasion assay was conducted

As the results above indicate HUVEC could suppress AR expression and our previous data showed that AR function as suppressor of autophagy in AR positive prostate cancer cells [14], we are curious whether HUVEC could induce autophagy directly and drugs targeting autophagy could attenuate cell invasion. So we stably express GFP-LC3 fusion protein in CRW22Rv1 cell then analyzed LC3II puncta number under fluorescence microscope. The number of puncta significantly increased in CRW22Rv1 when coculturing with HUVEC. Even AR was activated by DHT, which may strongly suppress autophagy, there are still plenty of LC3II puncta remained in cells (Fig. 3a). As p62 was directly regulated by AR [14], western blot showed that AR expression was decreased with concordantly p62 expression fell off (Fig. 3b). Surprisingly, LC3II expression elevates, indicating increased autophagy flux. DHT treatment significantly induced AR expression and consequently LC3II going down (Fig. 3b). We speculate that HUVEC possibly modulates autophagy through AR signaling. To confirm this, we knocked down AR expression by transfecting prostate cancer cells with siRNA targeting AR. The level AR was confirmed by western blot (Fig. 3c). As a result, AR knockdown resulted in p62 decreasing and LC3II enhancement; while the effect of DHT on prostate cancer cells was eliminated in AR knockdown cells (Fig. 3c, d). The above results were validated in AR overexpression PC-3 cells as AR activation strongly suppressed LC3II and HUVEC coculturing partially rescued LC3II (Additional file 4: Figure S3A). Atg5 is an autophagy-regulatory protein required for autophagosome formation. We critically inhibited autophagy by knocking down ATG5 expression to test the mechanism of HUVEC in inducing autophagy. The expression of Beclin-1, which has a central role in autophagy, was examined to track the autophagy signaling change. HUVEC coculturing didn’t change expression of Beclin-1 and Atg5. Rapamycin is an inducer of autophagy that inhibits of mTOR pathway. Treating cells with 10 μg/ml rapamycin increased Atg5, Beclin-1 and LC3-II. Knockdown of Atg5 resulted in the absence of LC3II, even at low AR level or in presence of rapamycin, indicating that the regulation effect of AR autophagy locates upstream of Beclin-1-ATG5 axis (Fig. 3e). At last, we blocked the autophagy flux by treating cells with chloroquine (CQ) in the coculture system to explore whether autophagy is required for metastasis of prostate cancer. And the result showed that CQ inhibited cell invasion without affecting cell viability (Fig. 3f, Additional file 4: Figure S3B). To summary, these data suggest that autophagy induced by endothelial cells may initiate tumor metastasis of prostate cancer.

### Autophagy induced by endothelial cells destabilized Paxillin and promotes cell motility

**Fig. 4:**
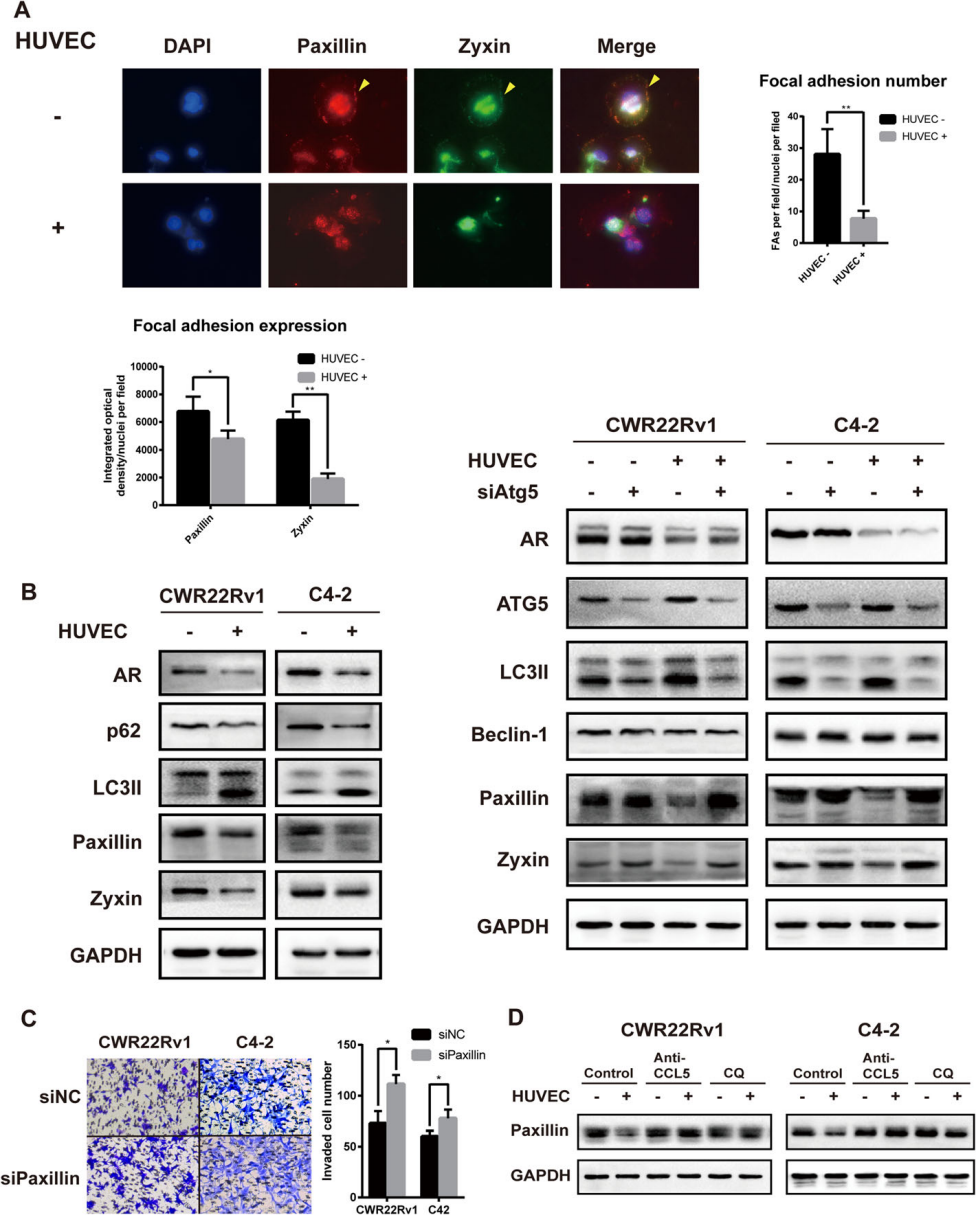
Autophagy induced by endothelial cells accelerates FAs disassembly and consequently promote cell invasion. **a** Immunofluorescence for Paxillin and Zyxin in CWR22Rv1 cell cultured with or without HUVEC. **b** Western blot analysis when prostate cancer cells cocultured with HUVEC and/or transfected with Atg5 siRNA. **c** C4–2 and CWR22Rv1 cells were transfected with siRNA target Paxillin or negative control siRNA and invasion assay was conducted. **d** Western blot analysis of Paxillin in cells treated with anti-CCL5–neutralizing antibody or CQ

Focal adhesions (FAs) are sites where integrin and proteoglycan mediated adhesion link the actin cytoskeleton to the extracellular matrix (ECM). The rate of cell migration is determined by turnover of FAs [15]. Paxillin and zyxin are important FA proteins, and paxillin plays a pivotal role as a scaffold at focal adhesions. Tyrosine phosphorylation of paxillin acts to reduce haptotactic cell migrations as well as transcellular invasive activities [16]. The most recent study shows that accumulation of paxillin in autophagy-deficient tumor cells impairs migration ability of motile cells, and co-localization of paxillin with autophagosome indicates paxillin is degraded by autophagy [17]. In this study, we did IF staining of paxillin and zyxin in CWR22Rv1 cell. The cells cocultured with HUVEC shows reduced FAs formation compared with single cultured cells (Fig. 4a). Western blot analysis shows that paxillin and zyxin are negatively related with LC3II, indicating the reported regulatory effect of autophagy on FAs also exists in prostate cancer cells (Fig. 4b). We further inhibited autophagy flux by knocking down the expression of Atg5. As expected, impaired autophagy resulted in accumulation of FAs proteins and reduced the function of HUVEC on prostate cancer cells (Fig. 4b). To determine whether paxillin levels underlie the cell motility, we used siRNA to knock down paxillin expression. As a result, reducing paxillin level increased number of invaded cells (Fig. 4c). Since AR directly regulates autophagy and CCL5 could suppress AR expression, we tested the effect of CCL5 on autophagy. CCL5 treatment induced expression of LC3II (Additional file 5: Figure S4). So we further treated cells with CCL5 neutralizing antibody or CQ to block the effect of HUVEC. Both ways are effective to rescue the paxillin level, indicating potential drug targets for tumor metastasis (Fig. 4d). Together, these results demonstrate that autophagy increase cell motility by increasing FAs disassembly.

### Combination of maraviroc and chloroquine showed efficacy of inhibiting metastasis in vivo

**Fig. 5:**
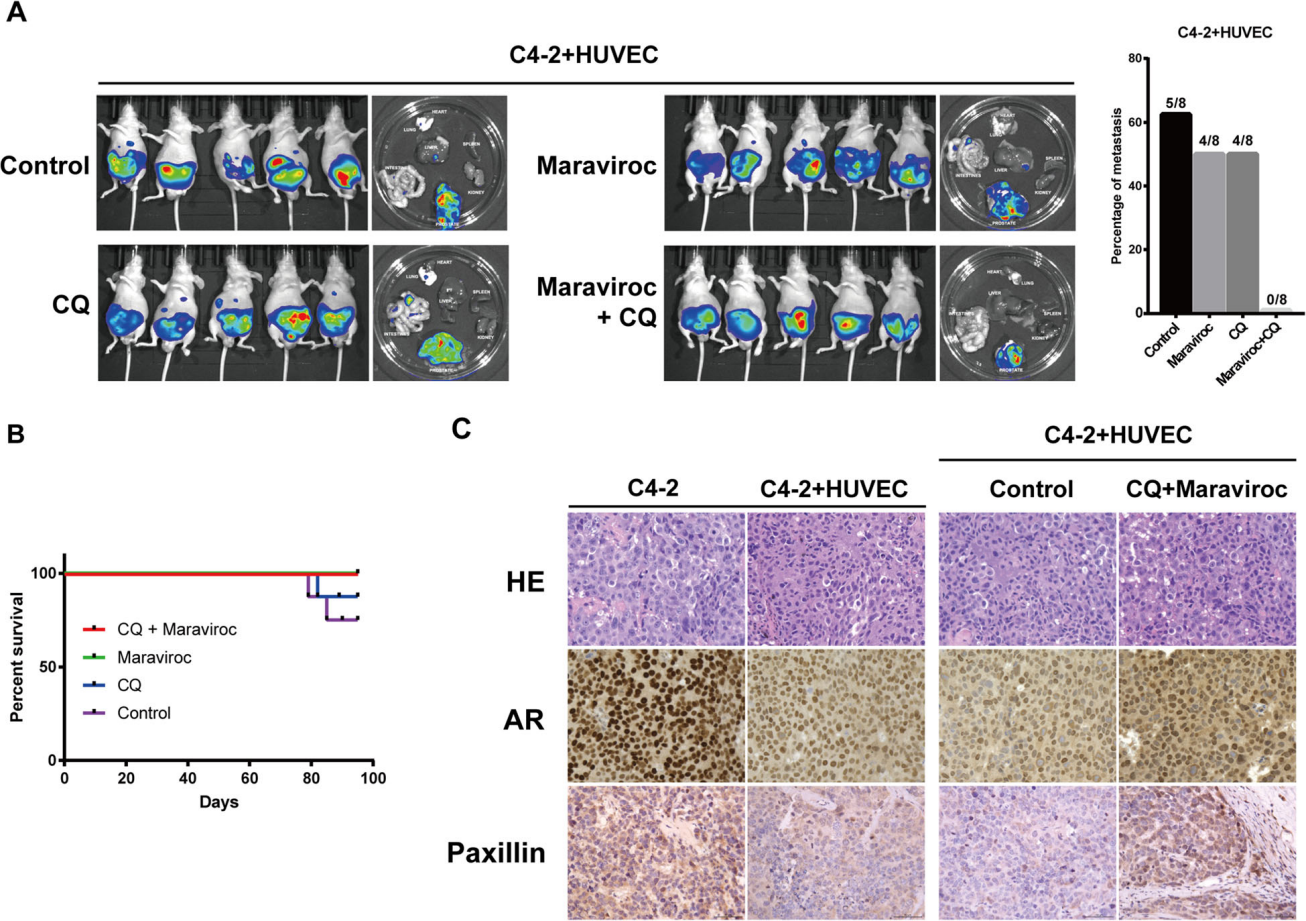
Drugs targeting CCL5/CCR5 and autophagy reduce metastasis in vivo. **a** In vivo bioluminescence imaging monitoring metastasis in orthotopic xenograft models. C4–2 cells was coimplanted with HUVEC. Mice were treated with CQ or Maraviroc alone or CQ + Maraviroc combination. **b** Overall survival of mice at the end of experiment. **c** Immunohistochemistry staining of AR and Paxillin in xenografts

Prostate cancer orthotopic xenograft model has been introduced for more than 20 years. It allows for the investigation of tumorigenic and metastatic processes and shows a high degree of lung and lymph node metastasis [18]. In view of the in vitro findings that CCL5/CCR5 signaling and autophagy play an important role in cell invasion, we used orthotopic xenograft models to explore the effect of CCL5/CCR5 and autophagy inhibition on the metastasis potential of prostate cancer. Give that metastasis incidence of prostate cancer cells coimplanted with HUVEC significantly is increased in orthotopic model [6], we initiated in vivo drug study. C4–2 was mixed with HUVEC and orthotopicly injected into dorsal lopes of mice prostate. Mice were treated with CQ or Maraviroc alone or CQ + Maraviroc combination. Control group were fed with PBS. By using bioluminescence imaging we monitored tumor metastasis every week. We detected tumor metastasis in control and CQ group 5 weeks after tumor implantation. On day 70, 62.5% (5/8) of the mice in control group, 50% (4/8) of the mice in Maraviroc group and 50% (4/8) of the mice in CQ group developed metastatic lesion (Fig. 5a). At the end-point of the experiment, all the mice in combination group survived, while 2 mice died at day 79 and day 85 respectively in control group (Fig. 5b). Immunohistochemistry demonstrated that both AR and paxillin expression decreased comparing coimplantation C4–2 with HUVEC with C4–2 alone. The drug study showed that CQ + Maraviroc rescued both AR and paxillin expression (Fig. 5c). CQ and Maraviroc treatment also successfully reduced autophagy level in vivo (Additional file 6: Figure S5A). H&E staining of organs (Additional file 6: Figure S5B) and mice body weight (Additional file 6: Figure S5C) suggest that combination treatment did not cause major toxicities. These data indicate that CQ combined with Maraviroc is effective to reduce metastasis by restoring paxillin expression.

## Discussion

In our previous study we demonstrated that the number of endothelial cells increased in prostate cancer compared normal tissues. Furthermore, castration or ADT treatment may also finally increase the microvascular density in the CRPC tumors [6]. The increasing infiltration of neovascular in tumors suggest that neovascularization plays critical roles in supplying nutrients for continuous tumor growth but also in providing cancer cells the access to the blood stream for distant metastasis [19]. We confirmed that endothelial cell is an important component of tumor microenvironment in promoting the metastatic potential of prostate cancer both in vitro and in vivo. The action of endothelial cells by enhancing the metastatic activity of prostate cancer was via repressing both AR expression and AR transcriptional activity. This is consistent with the previous studies [20, 21], though some studies still indicating the positive role of AR in promoting prostate cancer metastasis [22, 23].

Both CCL5 and its CCR5 are expressed in prostate cancer cells [24]. Increased surrounding infiltrating bone marrow mesenchymal stem cells directly suppress AR expression through CCL5/HIF2α pathway and CCL5 from the bone microenvironment has been shown to promote the growth of prostate cancer bone metastases [25]. Here, as CCL5 is the most significantly secreted factor by endothelial cells, we are the first to demonstrate CCL5 may be one of the key factors contributing to improving invasion of prostate cancer cells. AR expression may also be downregulated by either endothelial cells or CCL5. Blocking the interaction between CCL5 and prostate cancer cells in context of endothelial cells can attenuate this effect. However, we failed to reduce metastases in mice by using single CCR5/CCL5 antagonist Maraviroc, which may be the multiple stimulation of TME that contributes to tumor progression.

To further study the mechanism of endothelial cells and CCL5 promoting cell invasion, we focused our efforts on autophagy as our previous study showed that AR repression induced autophagy. High-throughput sequencing and microarray data in multiple clinical cohorts showed that AR activity is decreased in metastatic lesion, but autophagy gene signature is increased [26]. These results provide strong evidence that negative regulation of autophagy by AR may play a pivotal role in prostate cancer metastasis. Macro-autophagy (hereafter autophagy) is a highly conserved catabolic process that targets cellular contents to the lysosomal compartment for degradation. Cells depend on autophagy pathway to turnover damaged organelles, pathogens and large protein aggregates as autophagy has the ability to degrade very large structures [27]. Autophagic degradation acts as an important source of amino acids, nucleotides and fatty acids, that has a complex and highly context-dependent role in tumorigenesis [28]. Autophagy appears to have contrasting roles depending on context such as disease stage [26]. Genetic engineering mouse models studies demonstrated that autophagy plays as tumor suppressor [29, 30], but autophagy is also necessary for maintenance and progression of the disease [31–33], as many cancers exhibit increased autophagy during progression. Recent studies indicate that autophagy get involved in multiple steps in the metastatic cascade of tumors [34].

Focal adhesion kinase (FAK) localizes at sites of cell adhesion to the extracellular matrix (ECM) and plays an important role in cellular migration and adhesion in both normal and cancer cells [35]. Paxillin (PXN) is one of the major components in FAK signaling. In prostate cancer, PXN acts as metastatic metastasis suppressor gene [36]. Autophagy has a direct role in focal adhesion dynamics. Various proteins including PXN, VCL (vinculin) and ZYX (zyxin) are observed to colocalize with GFP-LC3 in migrating cells. Sharifi and colleagues identified PXN degradation is facilitated by direct interaction with LC3 [17]. By co-immunoprecipitation, they found LIR motif of PXN protein is responsible for PXN- LC3 binding. And cell motility defects in autophagy-deficient cells are due to the inability to degrade PXN [17]. In our study, we validate that endothelial cell induced autophagy reduced PXN and ZYX expression in AR positive prostate cancer cells. The epithelial to mesenchymal transition (EMT) regulator, Twist was reported to been bound to autophagy cargo adapter p62/Sqstm1, leading to decrease proteasomal degradation and increased EMT [37]. However, we didn’t observed changes of any EMT markers or regulators including E-cadherin and TGF-β (data not shown). As p62 has been shown to transcriptionally regulated by AR and suppress autophagy in prostate cancer, we found the expression of p62 was reduced when coculturing with endothelial cell, followed by LC3B induction. Restoration of p62 expression in context of HUVEC coculturing suppressed autophagy, consequently accumulation of PXN and ZYX. We also found that reducing PXN levels restores both focal adhesion morphology and motility, confirming the results in mammary cancer. To validate the in vitro data, we perform the drug treatment in orthotopic murine model. Although previous study showed inhibition effect of Maraviroc or autophagy inhibitor CQ on tumor metastasis [38, 39], our single drug treatment with CQ or Maraviroc showed little effect on repressing tumor metastasis. As cancer cells are surrounded tumor microenviroment, various components of tumor microenviroment contributes induction of autophagy. Hypoxia, anoxia, nutrient deprivation and inflammation are master factors play a role in autophagy initiation in tumors, consequently promote develop and metastasis of prostate cancer [40]. Thus single drugs that only target cytokine pathway or single autophagy pathway is insufficient. The combination drug treatment significantly reduced metastatic lesion and improved overall survival of mice, indicating an effective drug combination for mCRPC treatment.

## Conclusions

To summary, our findings will give further insights into the promotion role of endothelial cells in prostate cancer metastasis as a component of the TME. CCL5 secreted by endothelial cells may act as the driver of tumor metastasis. We also emphasize the importance of autophagy in prostate cancer progression. Drugs targeting both tumor endothelial cells and autophagy may be promising alternative choices treating mCRPC. Further clinical trials are expected to confirm our results and finally benefit the patients.

## Additional files

Additional file 1: **Table S1**. (XLSX 10 kb)

Additional file 2: **Figure S1.** Cell viability assay for effect of coculturing with HUVEC. (TIF 210 kb)

Additional file 3: **Figure S2. A**. qPCR validation of CCL5 in prostate cancer cells or HUVEC after transfecting siCCL5 for 72 h; **B**. CCL5 concentration in coculture media tested by ELISA. (TIF 246 kb)

Additional file 4: **Figure S3. A**. Western blotting of PC-3 and PC-3-AR cells and quantification of LC3II/LC3I ratio; **B**. Cell viability assay for effect of CQ treatment. (TIF 716 kb)

Additional file 5: **Figure S4.** Effect of CCL5 treatment on autophagy by western blotting. (TIF 214 kb)

Additional file 6: **Figure S5. A**. Western blotting of xenograft tumors; **B**. HE staining of mice organs; **C**. Mice weight variance. (TIF 3137 kb)

## Abbreviations

ADT: Androgen-deprivation therapy
AR: Androgen receptor
ARE: Androgen response element
CCL5: C-C motif chemokine ligand 5
CCR5: C-C motif chemokine receptor 5
CPRC: Castration-resistant prostate cancer
CQ: Chloroquine
DHT: Dihydrotestosterone
ECM: Extracellular matrix
EMT: Epithelial to mesenchymal transition
FA: Focal adhesion
FAK: Focal adhesion kinase
H&E: Hematoxylin and eosin
HUVEC: Human umbilical vein endothelial cells
IF: Immunofluorescence
mCPRC: metastatic castration resistant prostate cancer (mCRPC)
MMTV: Mouse mammary tumor virus
TME: Tumor microenvironment
