# Supplementary figures and images for: Endothelial cells promote metastasis of prostate cancer by enhancing autophagy
Source: J Exp Clin Cancer Res. 2018 Sep 10;37:221. doi: 10.1186/s13046-018-0884-2 (PMC6131784; doi:10.1186/s13046-018-0884-2)

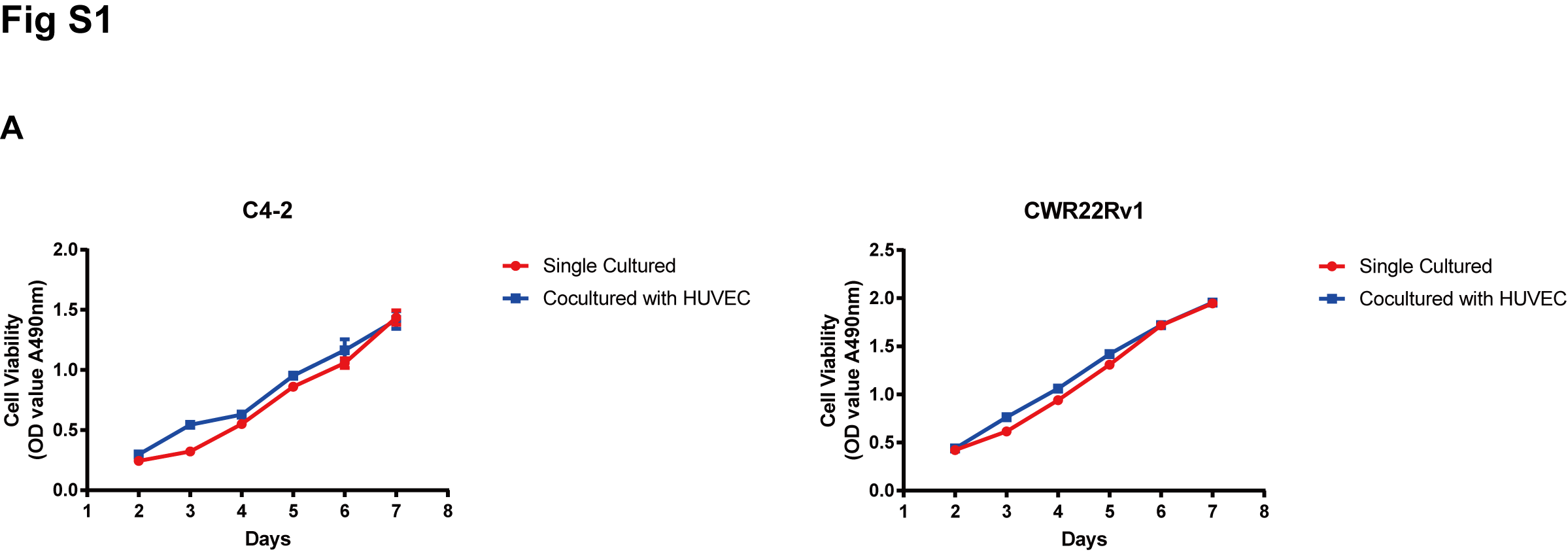

Supplement: Supplementary file 2 — Figure S1. Cell viability assay for effect of coculturing with HUVEC. (TIF 210 kb) [file 13046_2018_884_MOESM2_ESM.tif]

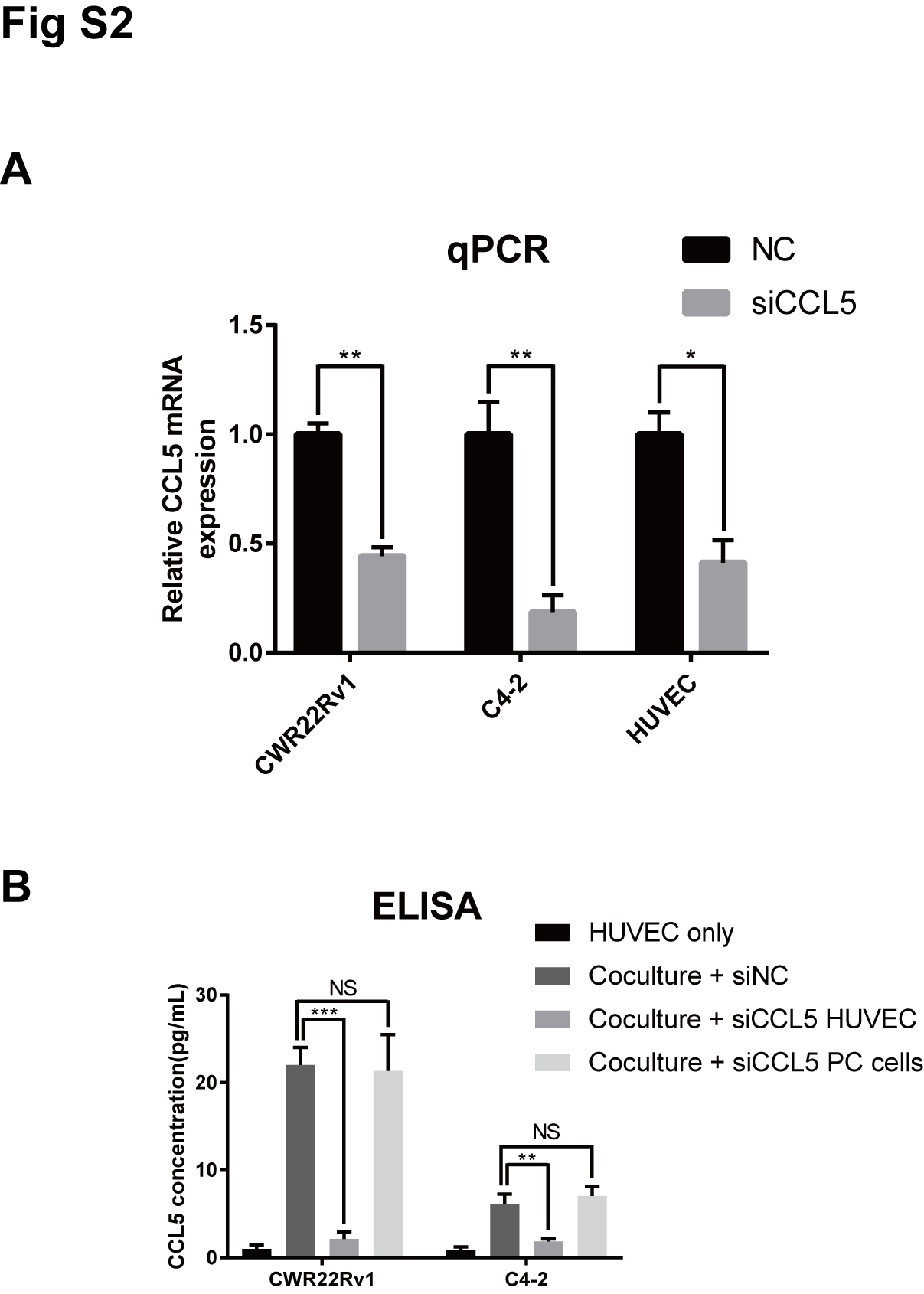

Supplement: Supplementary file 3 — Figure S2. A. qPCR validation of CCL5 in prostate cancer cells or HUVEC after transfecting siCCL5 for 72 h; B. CCL5 concentration in coculture media tested by ELISA. (TIF 246 kb) [file 13046_2018_884_MOESM3_ESM.tif]

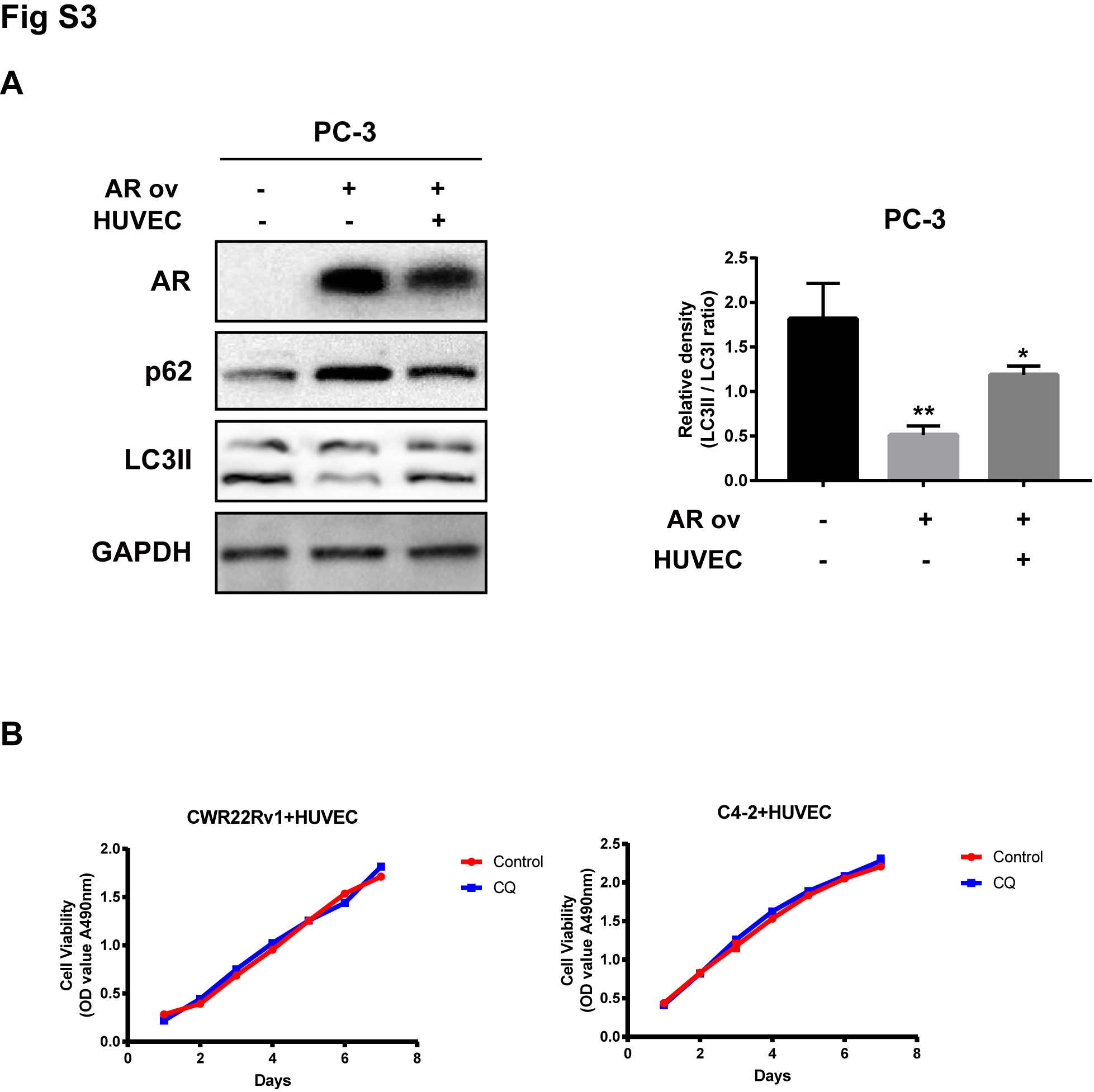

Supplement: Supplementary file 4 — Figure S3. A. Western blotting of PC-3 and PC-3-AR cells and quantification of LC3II/LC3I ratio; B. Cell viability assay for effect of CQ treatment. (TIF 716 kb) [file 13046_2018_884_MOESM4_ESM.tif]

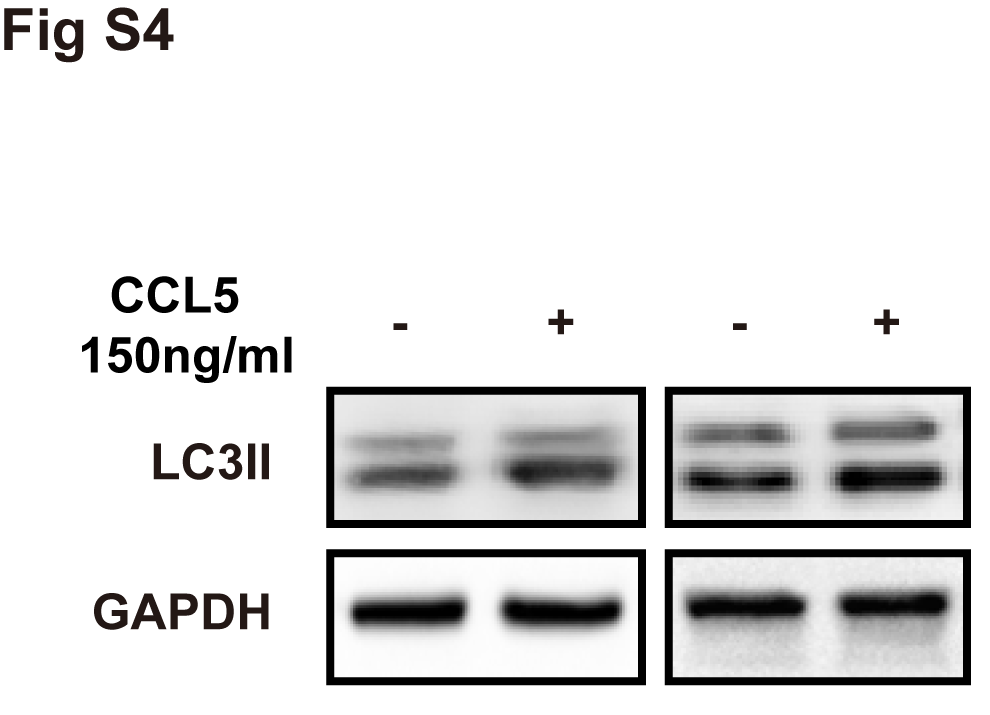

Supplement: Supplementary file 5 — Figure S4. Effect of CCL5 treatment on autophagy by western blotting. (TIF 214 kb) [file 13046_2018_884_MOESM5_ESM.tif]

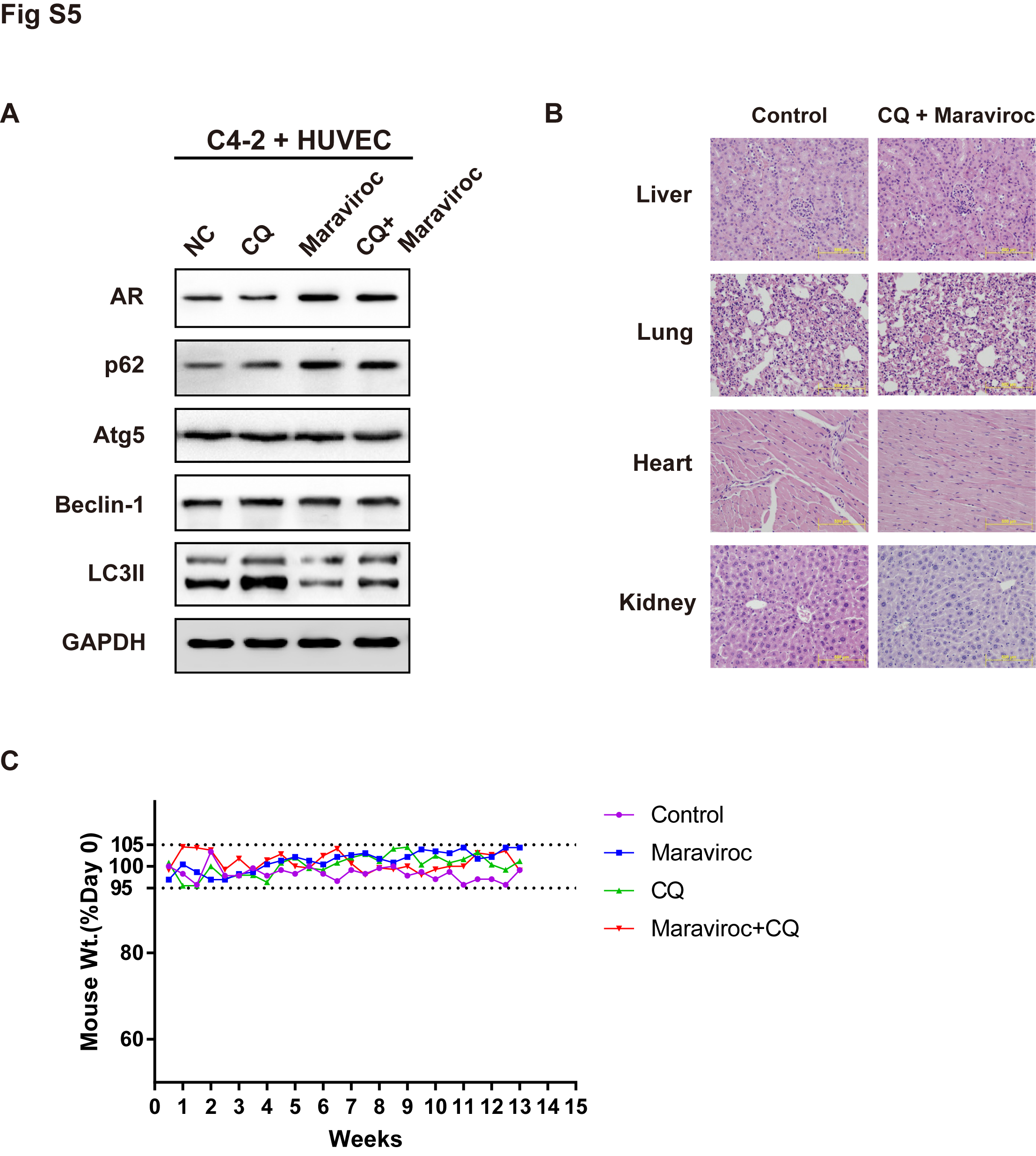

Supplement: Supplementary file 6 — Figure S5. A. Western blotting of xenograft tumors; B. HE staining of mice organs; C. Mice weight variance. (TIF 3137 kb) [file 13046_2018_884_MOESM6_ESM.tif]
